# Investigating the impact of London’s ultra low emission zone on children’s health: children’s health in London and Luton (CHILL) protocol for a prospective parallel cohort study

**DOI:** 10.1186/s12887-023-04384-5

**Published:** 2023-11-04

**Authors:** Ivelina Tsocheva, James Scales, Rosamund Dove, Jasmine Chavda, Harpal Kalsi, Helen E Wood, Grainne Colligan, Louise Cross, Chris Newby, Amy Hall, Mia Keating, Luke Sartori, Jessica Moon, Ann Thomson, Florian Tomini, Aisling Murray, Wasim Hamad, Sarah Tijm, Alice Hirst, Britzer Paul Vincent, Pavani Kotala, Frances Balkwill, Borislava Mihaylova, Jonathan Grigg, Jennifer K Quint, Monica Fletcher, Mark Mon-Williams, John Wright, Esther van Sluijs, Sean Beevers, Gurch Randhawa, Sandra Eldridge, Aziz Sheikh, William Gauderman, Frank Kelly, Ian S Mudway, Christopher J Griffiths

**Affiliations:** 1 Institute for Health Research, University of Bedfordshire, Putteridge Bury, Hitchin Road, Bedfordshire, LU2 8LE UK; 2 Asthma UK Centre for Applied Research, London, UK; 3 Wolfson Institute of Population Health, Faculty of Medicine and Dentistry, Queen Mary University of London, London, UK; 4 University of Nottingham, Nottingham, UK; 5 Centre of the Cell, Queen Mary University of London, London, UK; 6 Imperial College, London, UK; 7 Usher Institute, University of Edinburgh, Edinburgh, UK; 8 Bradford Institute for Health Research, Bradford, UK; 9 MRC Epidemiology Unit, University of Cambridge, Cambridge, UK; 10 MRC Centre for Environment and Health, Imperial College London, London, UK; 11 MRC - Asthma UK Centre in Allergic Mechanisms of Asthma, London, UK; 12 Keck School of Medicine, University of Southern California, Los Angeles, USA; 13 NIHR Health Protection Research Unit in Environmental Exposures and Health, Imperial College London, London, UK

**Keywords:** Children, Cohort, Ethnicity, Air pollution, Lung function, Health impacts, Health inequalities, Costs

## Abstract

**Background:**

Air pollution harms health across the life course. Children are at particular risk of adverse effects during development, which may impact on health in later life. Interventions that improve air quality are urgently needed both to improve public health now, and prevent longer-term increased vulnerability to chronic disease. Low Emission Zones are a public health policy intervention aimed at reducing traffic-derived contributions to urban air pollution, but evidence that they deliver health benefits is lacking. We describe a natural experiment study (CHILL: Children’s Health in London and Luton) to evaluate the impacts of the introduction of London’s Ultra Low Emission Zone (ULEZ) on children’s health.

**Methods:**

CHILL is a prospective two-arm parallel longitudinal cohort study recruiting children at age 6–9 years from primary schools in Central London (the focus of the first phase of the ULEZ) and Luton (a comparator site), with the primary outcome being the impact of changes in annual air pollutant exposures (nitrogen oxides [NOx], nitrogen dioxide [NO_2_], particulate matter with a diameter of less than 2.5micrograms [PM_2.5_], and less than 10 micrograms [PM_10_]) across the two sites on lung function growth, measured as post-bronchodilator forced expiratory volume in one second (FEV_1_) over five years. Secondary outcomes include physical activity, cognitive development, mental health, quality of life, health inequalities, and a range of respiratory and health economic data.

**Discussion:**

CHILL’s prospective parallel cohort design will enable robust conclusions to be drawn on the effectiveness of the ULEZ at improving air quality and delivering improvements in children’s respiratory health. With increasing proportions of the world’s population now living in large urban areas exceeding World Health Organisation air pollution limit guidelines, our study findings will have important implications for the design and implementation of Low Emission and Clean Air Zones in the UK, and worldwide.

**ClinicalTrials.gov:**

NCT04695093 (05/01/2021).

**Supplementary Information:**

The online version contains supplementary material available at 10.1186/s12887-023-04384-5.

## Background

Traffic-related air pollution is associated with adverse health effects across the life course and substantial health inequalities [1, 2]. Children are particularly vulnerable, [1, 3] with adverse effects observed on developmental trajectories and long-term health, including birth outcomes [4] stunted lung growth, [5] delayed cognitive development [6] and increased incidence of psychiatric disorders [7]. Exposure to air pollution during infancy and childhood is associated with increased risk in later life of asthma, pneumonia and chronic obstructive pulmonary disease [8]. Even exposures below legal limit values are associated with disability, disease and death in childhood [1].

Cohort studies have played a key role in identifying these impacts. The ESCAPE meta-analysis of five European birth cohorts showed poor air quality was associated with reduced lung function in pre-adolescent children [9]. The California Children’s Health Study (CHS) showed clinically important deficits of lung growth and function in adolescents [10–13]. Whilst these studies have illustrated associations between adverse responses and air pollutant exposures, it is also notable that downward trends in air pollution have also been shown to deliver health improvements. This was illustrated by the successive children’s cohorts within the CHS (between 1994 and 2011) where the proportion of adolescents with clinically significant deficits in lung function fell as air quality in California improved [14].

There is therefore an urgent need to identify the most impactful policy interventions that improve air quality and deliver health benefits. Low Emission Zones (LEZ), which restrict the entry of polluting vehicles to urban areas, and related Clean Air Zones, have become the dominant public health policy intervention in the effort to improve air quality across Europe [15]. To date, studies evaluating their impact have shown variable effects on road use, [16] exhaust emissions, and air quality, with few identifying clear health benefits [5, 17–24]. A systematic review which evaluated the impact of air quality strategies across Europe on health and health inequalities found negligible effects on respiratory symptoms [23] with only one of 15 the studies identified gathered health data directly from individuals [24]. There was limited evidence for impact of LEZs on air quality in five EU countries (Denmark, the Netherlands, Germany, Italy and the UK) though the health impact of these changes were not addressed [25]. A recent Cochrane systematic review focusing on interventions to reduce ambient particulate matter air pollution and their effect on health identified few studies, with only low or very low grade evidence that interventions targeting vehicular sources improved air quality or health [26].

London implemented its first LEZ from 2008 to 2012, with phased tightening of emission standards in 2008 and 2012. We evaluated its impact, using a sequential cross sectional design, on the health of 2,297 east London primary school children, [5, 27] finding small improvements in air quality (most clearly NO_2_ reductions at the roadside), but no convincing health benefit, such as improvements in lung function or respiratory symptoms.[29] Furthermore, over the study period, we identified significant deficits in participating children’s lung capacity of between 5 and 10%, associated with exposures to traffic-related pollutants.

In 2019, London began implementing a second LEZ with more ambitious air quality targets, termed the ‘Ultra Low Emission Zone’ (ULEZ) [28]. Funded by NIHR Public Health Research, we established the Children’s Health in London and Luton (CHILL) study, a natural experiment evaluation to determine whether the ULEZ improves air quality and children’s respiratory health (NIHR PHR 16/139/01). CHILL is a prospective two-arm parallel longitudinal cohort study recruiting children aged 6–9 years, attending primary schools in the central area of London (the focus of the ULEZ first phase) and Luton (a comparator site), with the primary outcome being the impact of annual air pollutant exposures on lung growth (measured as FEV_1_ and FVC, post bronchodilator), over four consecutive years. These objectives were further enhanced prior to the initiation of the study to consider the impacts of the ULEZ implementation on physical activity, travel behaviours and obesity using funding from the NIHR funded Collaboration for Leadership in Applied Health Research and Care (CLAHRC) and the Applied Research Collaboration (ARC), North Thames.

By collecting sequential annual lung function, respiratory symptoms data and information on health care use from participants at both locations and relating these to annual and monthly modelled exposures to criterion air pollutants: nitrogen dioxide (NO_2_), ozone (O_3_) and particulate matter (both PM of less than 5 and 10 microns, PM_2.5_ and PM_10_ respectively) over the duration of the study, the proposed work will provide valuable insights on the effectiveness of air quality regulatory action on children’s health.

To compensate for impacts of the COVID-19 pandemic on data collection we will add a fifth year of data collection, following up participants moving to secondary schools, and assessing sero-positivity for SARS-CoV-2 antibodies.

CHILL meets all the criteria for a high-quality natural experiment evaluation including: prospective design, comparator site, large representative population sample, detailed air quality measurements, health record data, with the potential for downstream mechanistic assessments through additional funding initiatives. If successful, our findings will provide the evidence for cities in the UK, Europe and globally to implement equally robust and ambitious air pollution interventions, to achieve similar health benefits. CHILL opened to recruitment in June 2018 to capture a year of pre-ULEZ baseline data.

## Methods

### Primary research question

- Does the implementation of London’s ULEZ improve lung function growth trajectories in children of primary school age?

### Secondary research questions

- To what extent does ULEZ implementation improve including air quality, specifically reductions in the emissions from diesel vehicles?
- Does ULEZ implementation result in a reduction in respiratory and allergic symptoms, and respiratory infections in primary school aged children?
- Does implementation of the ULEZ encourage increased outdoor physical activity, alter travel behaviours and impact on children’s weight and risk of obesity?
- Does the ULEZ deliver measurable benefits in the perception of quality of life?
- Does the ULEZ reduce health inequalities, health care use and associated costs?
- Does SARS-Cov-2 infection affect children’s lung development?

### Outcome measures

#### Primary

Lung growth (post-bronchodilator forced expiratory volume in one second, FEV_1_).

#### Secondary

Air quality, forced vital capacity (FVC), respiratory symptoms, respiratory infections, physical activity, quality of life, health care use and costs, seroprevalence of SARS-CoV-2 antibodies, and a range of respiratory and health economic data.

Details of outcome measures and the timing and means of their collection are given in Table 1.

**Table 1:** Outcome Measures

Type	Outcome	Method	Year 1	Year 2	Year 3	Year 4	Year 5	Recording	Analysis
Primary	FEV_1_	Annual health assessment: spirometry	school visit	school visit	school visit	school visit	school visit	Spirometer download	Interaction analysis
Exposures	Air quality: NO, NO_2_, PM10, PM2.5	k-means clustering developed by Font et al.(27)	LAQN* and AURN**	LAQN and AURN	LAQN and AURN	LAQN and AURN	LAQN and AURN	LAQN and AURN	Interaction analysis
Secondary	FVC and other spirometric variables	Annual health assessment: spirometry	school visit	school visit	school visit	school visit	school visit	Spirometer download	Interaction analysis
Secondary	Physical activity and GPS tracking	Annual health assessment: accelerometer and GPS tracker	school visit	school visit	school visit	school visit	school visit	Accelerometer and GPS download	Interaction analysis
Demography / potential confounding variables		Parent-completed questionnaire: (paper or web)	school bag prior to school visit	school bag prior to school visit	school bag prior to school visit	school bag prior to school visit	school bag prior to school visit	Clinical Record Form	Interaction analysis
Secondary	School absence; work absence	Parent-completed questionnaire: (paper or web)	school bag prior to school visit	school bag prior to school visit	school bag prior to school visit	school bag prior to school visit	school bag prior to school visit	Clinical Record Form	Interaction analysis
Secondary	Respiratory and allergy symptoms (ISAAC^+^)	Parent-completed questionnaire: (paper or web)	school bag prior to school visit	school bag prior to school visit	school bag prior to school visit	school bag prior to school visit	school bag prior to school visit	Clinical Record Form	Interaction analysis
Secondary	QOL (CHU9D^++^)	Parent-completed questionnaire: (paper or web)	school bag prior to school visit	school bag prior to school visit	school bag prior to school visit	school bag prior to school visit	school bag prior to school visit	Clinical Record From	Interaction analysis
Secondary	Non-NHS costs	Parent-completed questionnaire: (paper or web)	school bag prior to school visit	school bag prior to school visit	school bag prior to school visit	school bag prior to school visit	school bag prior to school visit	Clinical Record From	
Secondary	Respiratory infection Health care use NHS costs	Data extraction from GP health records	Gathered after year 4 school visits complete	Gathered after year 4 school visits complete	Gathered after year 4 school visits complete	Gathered after year 4 school visits complete	Gathered after year 4 school visits complete	Clinical Record Form	Interaction analysis
Secondary	Health care use NHS costs	Data extraction from GP health records, HES^+++^ data linkage	n/a	n/a	n/a	n/a	Data extraction in year 5	Clinical Record Form/HES data	Interaction analysis
Secondary	SARS-CoV-2 antibodies	Finger-prick blood test	n/a	n/a	n/a	School visit	n/a	Clinical Record Form	Interaction analysis

* LAQN: London Air Quality Network

** AURN: Automatic Urban & Rural Network

^+^ ISAAC: International Study of Asthma and Allergy in Children Questionnaire

++ CHU9D: Child Health Utility 9D score

+++ HES: Hospital Episode Statistics

### Study design

The CHILL study is a prospective parallel longitudinal cohort study performed over 4 years at two separate locations, one impacted by the ULEZ road traffic management scheme and one not. The full study design is outlined in Additional file 1 -Fig. 1.

### Study population and setting

Children (age 6–9 years) are recruited from 44 London primary schools (years 2, 3, 4) with a catchment area within and bordering the Central London ULEZ area, and 41 primary schools (year 2, 3, 4) from the Boroughs of Luton and Dunstable. The Luton area is chosen as a suitable comparison site to Central London due to its broadly similar air quality, demography, and levels of socio-economic deprivation. Whilst it is subject to projected air quality improvements through national government policy, it does not have the ambitious local scale policies being enacted within London. It is also sufficiently distant from London to be free from risk of contamination by effects of the ULEZ. School and participant eligibility criteria are summarised in Table 2.

**Table 2:** Eligibility Criteria

	Primary schools	Individuals
**Inclusion criteria**	Any school located within, or whose catchment area includes the Central London ULEZ; or within the Boroughs of Luton and Dunstable.	All children attending a study school, in years 2, 3, or 4 at study inception.
**Exclusion criteria**	Primary schools that are not within the above boundaries outlined above.	Children with learning or physical disabilities sufficient for them to be unable to give informed assent to the study, or to carry out study procedures. Children with major lung disease (not including asthma).

### Study interventions

#### London ultra low emission zone

The Central London ULEZ, implemented on April 8th 2019 uses number plate recognition technology and a daily penalty charge notice issued for vehicles entering this central zone not meeting the set standards – as outlined in Additional File 2 -Fig. 2 It superseded the previous T-charge, which set minimum emission standards of Euro 4/IV for petrol and diesel vehicles and Euro 3 for motorised tricycles and quadricycles, increasing the emission standards to Euro 6 for diesel vehicles. It applies to all vehicles, 24 h a day across the whole year, except for Christmas day. The ULEZ was subsequently extended to the North Circular Road (A406) and South Circular Road (A205) in October 2021 (orange zone), with a London-wide ULEZ for heavy duty vehicles (HDV) within the current LEZ boundary. As a consequence of the first wave of the Corona virus outbreak in the UK, the ULEZ and the Central London Congestion Charging Zone were suspended between the 23rd March − 18th May 2020.

#### Luton: comparison site

Luton’s air quality is influenced by several factors: the presence of major industry (including a motor industry), the transecting M1 motorway and major road A505, and a rapidly expanding international airport, all bringing significant traffic flows into and through the town. Luton has no plans for a Clean Air Zone. It has three designated Air Quality Management Areas. Planned interventions for air quality improvement include a busway, car sharing, public information and advice systems, and provision of charging points for electric vehicles.

### Sample size

We use 90% power and 0.05 significance level to test a 15 ml difference in FEV_1_ growth per year between Central ULEZ and Luton comparison cohorts. For 40 schools/arm with 40 children from any of school years 2, 3, or 4, the study is powered for 15ml per year difference in FEV_1_ growth between the comparison zone and ULEZ. The total target sample size is therefore 3,200 children, comprising 1,600 in the London cohort and 1,600 in the Luton cohort.

### Assumptions

- Adjustment for clustering of lung function outcomes within schools, by inflating sample size using an intra-class correlation (ICC) for FEV_1_ in schools calculated from our original ULEZ study (ICC = 0.001).
- 70% success rate in children in classes Y2 and Y3 for a valid reading for FEV_1_.
- 20% attrition per year of follow up, reflecting children moving schools or withdrawing.
- 30% inflation to enable subgroup analysis.

With COVID-19 lockdowns closing schools and leading to loss of data collection during study years 2 and 3, we calculate that addition of a 5th year of data collection with follow up of children moving to secondary schools during study years 4 and 5 will deliver study power as follows:

- 25% capture of movers to secondary schools retains 80% power.
- 50%, 75% and 100% capture of movers retains over 90% power.

### Recruitment

#### School recruitment

All schools meeting the inclusion criteria are invited to take part and made aware of the study through local media and contact with local leaders and parent groups. An initial invitation email with a link to a short YouTube video summarising the study is sent to headteachers of all schools, [29] followed up by a call from the study Chief Investigator.

#### Child recruitment and informed consent

Informed consent forms are completed by caregivers and children at home, and require opt-in to study components, including elements of the health assessments and access to GP health records and Hospital Episode Statistics (HES) data. The recruitment and informed consent procedure follows a “school bag” approach, facilitated by school staff, Additional file 3 -Fig. 3. Study documents include a QR code link to the study video.

### Patient and public involvement

Patient and public involvement (PPI) in the CHILL study is integral to the study and makes both a formal and informal contribution. Study design is informed by consultation with parents, headteachers, children from the study areas, and community pressure groups such as ‘Mums for Lungs’.

The CHILL Study, as a progression of previous research projects, [5] benefits from an established network of interested public, forming the CHILL study dedicated PPI group. The group aims to ensure that the perspectives and welfare of the participant children, care givers and schools remain at the centre of the study throughout. The PPI group provides: (i) comment and advice on study materials; (ii) supports recruitment and retention in the study; (iii) advises on dissemination of progress and findings and (iv) provides representatives who are members of the Project Management Group (PMG) and Independent Steering Committee (ISC).

Informal PPI between the study team and school staff, children and caregivers is ongoing throughout the lifetime of the project.

### Science outreach education

Central to the CHILL study community outreach strategy is to engage children from participating schools as active participants in science and health research. Most study schools are in areas of socioeconomic deprivation with high proportions of families from Black, Asian and ethnic minority (BAME) heritages. To this end, an interactive science outreach session addressing air pollution and health is delivered by a learning outreach officer to all school classes participating in the study, regardless of how many individual children are taking part in health assessments. Children and class teachers are invited to provide feedback on the sessions to be taken into consideration for future science session planning and involvement. The intention is each year to deliver a fresh outreach session addressing a different aspect of air pollution and health that links to the CHILL core respiratory study, and genetics and cognitive development sub-studies:

- Study year 1: Air pollution and your lungs.
- Study year 2: Air pollution and your genes.
- Study year 3: Online outreach education resources available to schools.
- Study year 4: Air pollution, your brain and cognition.
- Study year 5: CHILL: Air pollution and action for the future.

### Data collection methods

Using different formats and measurement tools (Table 2), data is collected in two ways: (1) directly from children and teachers at health assessments during school visits and (2) from the annual study questionnaire filled out by caregivers returned *via* the school bag approach.

### Annual health assessments

Annual health assessments take place at each school as far as possible during the same month of each year. The study team assess children in groups of four or five in a suitable room identified by the class-teacher, using standard protocols. Assessments take about 35 min per child and include:

- Height, weight, body mass index (BMI).
- Pre- and post-bronchodilator spirometry.
- Annual study questionnaire for home completion by parents/guardians.

#### Spirometry

Following height (sitting and standing) and weight measurements, lung function is measured before and 15 min after bronchodilation, according to European Thoracic Society guidelines [30] by fully trained members of the study team using a Vitalograph 6000 Alpha Touch Spirometer, [31] calibrated using three litre precision syringe at the start of each session. Children inhale four puffs (100mcg/puff) of salbutamol *via* a large volume spacer, administered by a study team member. A new or sterilised spacer is used for each child.

#### Annual study questionnaire for home completion

Caregivers are asked to complete questionnaires in year 1 (when informed consent given to the study) and in study years 2, 3, 4 and 5. These comprise.

- Demography and residential history.
- Parent-reported respiratory and allergy symptoms (ISAAC questionnaire) [32].
- Parent-reported paediatric quality of life (CHU9D questionnaire) [33].
- Non-NHS costs.
- Child absence from school.
- Smoking/exposure to second-hand smoke during pregnancy.
- Travel choices.
- Parental absence from work due to child ill-health.
- Upon receipt of a completed questionnaire, caregivers receive a £5 supermarket voucher.

### Additional assessments

With additional funding, the core study is being extended with additional assessments taking place during the annual health assessment. Informed consent is obtained through the same school bag approach used in the core study.

*Physical activity* (study years 1, 2, 3 and 4): Each child is fitted with an Actigraph accelerometer and provided with instructions (verbal and paper) on its use. Children are requested to wear the monitor during waking hours for 7 days after which they are collected from the school. Children answer a question on their route to school and means of transport on the day of assessment. Children in some schools have the option of wearing a GPS (Global Positioning Satellite) monitor for a week to track the routes they take to and from school.

*Capillary blood test for SARS-CoV-2 antibodies and heavy metals* (study year 4 and 5): Children with informed consent provide a finger-prick capillary blood sample for testing on a Fortress COVID-19 rapid antibody lateral flow test cassette, the coating antigen for which is recombinant Spike-RBD-Human Fc fragment [34]. Children are given a letter to take home giving their antibody test result, its interpretation, and advice to follow current UK Government COVID-19 guidance.

### NHS health records

Following approval by an NHS research ethics committee and with participants’ informed consent we are approaching the Caldicott Guardians of participants’ general practices or the relevant local primary care health care record data service to request electronic downloads of coded electronic health record data from birth to current age, addressing respiratory, mental health, COVID-19 testing and vaccination status, and health care use. Data collection takes place during year 4 and 5 of the study.

### Air pollution modelling

Monthly exposure estimates will be produced at 20m^2^ resolution over the study domains using the CMAQ (The Community Multiscale Air Quality Modelling System)-urban model which couples the Advanced Dispersion roads Model (ADMS) with the Weather Research and Forecasting model WRF meteorological model and CMAQ regional scale models. A comprehensive description of the CMAQ-urban model has been published previously [35–37] and will be employed in the present study to model NOx, NO_2_, O_3_, PM_2.5_, PM_10_, PM (Primary Organic Aerosol (POA), Secondary Organic Aerosol (SOA), nitrate, sulphate, black carbon), as well as exhaust and non-exhaust PM contributions over the period 2018–2020. Model outputs will be provided at residential address level.

### Data protection and management

Participants are allocated a unique study ID number. Data collection, confidentiality, entry, security and storage and protection is managed according to principles of good clinical practice and in line with our Clinical Trials Unit protocols.

Data handling and record keeping are overseen by the Pragmatic Clinical Trials Unit (PCTU) based at QMUL. The Data Manager has developed appropriate data management strategies for the study and advises on their implementation. Advice is provided on current regulatory framework regarding data protection and data management procedures in compliance with the Data Protection Act and trial regulations. All databases have integrated data validation checks and audit trails. The PCTU data management team advise on electronic data security. They will also advise on data transfer, storage, back-up and archiving of data and ensure databases are regularly backed up and data safeguarded from accidental loss. Paper records, CRFs and informed consent forms and recruitment logs are held locally in line with governance procedures.

### Data

#### Statistical methods

The primary outcome variable of post bronchodilator FEV_1_, and secondary outcome variables of air pollution, post bronchodilator FVC and physical activity will be analysed longitudinally through a mixed effect model. The model assesses the interaction effect of time and study group to determine the difference in variable change between the intervention group (London) and comparison group (Luton). Mixed effects models using a random intercept term are robust to the biases of missing data and the clustering effect of recruiting participants from multiple schools, facilitating a longitudinal design in a highly transient population. Primary and secondary outcome variables will be adjusted to account for known covariates of gender, asthma diagnosis, ethnicity, deprivation, [38] age, height, and BMI. Secondary covariates which may be included after sensitivity analysis include socioeconomic status, stress, and physical activity. For linking air pollution data with lung function over time, a more complex mixed effect model with three levels will be used, the levels being child, school, and group. These will be tested first on a complete model with all levels, if models are not identifiable, then simpler models will be tried with fewer levels. These models would allow for the effects of each air pollutant to be tested against lung function at each level as well as over time.

#### Sensitivity analyses and missing data

Sensitivity analyses will be conducted to ensure no bias in the primary results due to missing data by contrasting an “all children” data set to a “complete history” data set. Temporal individual and school exposure assessments will be contrasted to the rest of the cohort to assess for cluster bias such as rebound lung growth and high non-traffic related air pollution change unrelated to the ULEZ.

Z-scores will be calculated for each continuous variable and will be examined for values > 4. Plots of residuals will be inspected for suspected outliers. Should any be found, raw data will be checked for accuracy. If the data are found to be correct, a model will be fitted excluding outliers as a sensitivity analysis to assess impact on the primary data set.

#### Additional analyses

Secondary analysis will assess the effect of the intervention on participants by quintile of deprivation, ethnic group and sex. Specifically, assessment will examine the following outcomes: change in air quality for each individual pollutant; lung growth; change in generic quality of life; change in respiratory symptoms; change in respiratory infection rates; change in health care use and change to small-area socioeconomic, population and mortality outcomes.

### Assessment of cost-effectiveness

An economic evaluation will relate costs associated with introducing the ULEZ to the impact of air pollution on children’s lung growth, school attendance, parent work productivity and use/costs of healthcare services and quality of life. Analysis will use a cost-consequences approach, as recommended in NICE (National Institute of Health and Care Excellence) public health guidance.

Downloads of each child’s electronic health record will provide data on primary and secondary care use, while unit costs from national sources will be applied to all resource use to estimate individual-level costs. Impacts of school absence will be assessed in relation to meeting government attendance targets and consequent lost productivity/income due to work absence by parents, using appropriate assumptions, national wage rate data and a human capital approach to costing. Quality of life and quality-adjusted life years will be assessed by administering the parent completed proxy Child Health Utility 9D and its associated general population-based preference weights. The cost-consequences analysis will report means and standard deviations for all costs and outcomes for both cohorts that will be assessed using a mixed effects model to assess interaction effects of time and study site.

### Ethics, safety and dissemination

The study is approved by Queen Mary University of London Research Ethics Committee (reference 2018/08), and NHS Research Ethics Committee (reference 22/WS/0065). The Chief Investigator has the overall oversight, responsibility and a duty to ensure that safety monitoring and reporting is conducted in accordance with the sponsor’s requirements and that an annual progress report, including any safety issues, is submitted yearly to the main research ethics committee and the sponsor. Six monthly progress reports are sent to the funder.

### Governance

An Independent Scientific Committee (ISC) comprising a chair, five scientific members and two PPI members monitors the study on behalf of the funder and sponsor. The ISC meets with the CI and study team at least annually.

### Data monitoring and auditing

Monitoring and auditing of data is carried out by the PCTU Quality Assurance team.

### Dissemination plan and project outputs

The CHILL study will disseminate study progress, maximising public and professional awareness of the study and its relevance to public and child health. We expect to reach the following groups:

- study participants, their families, schools and local study communities;
- the public - including voluntary organisations, charities, lay and pressure groups.
- government - including parliament, national, regional, local councils, International governments;
- academia – including universities, NIHR, Royal Colleges, National Institute for Health and Care Excellence (NICE), World Health Organization (WHO), and leading international research groups;
- industry – vehicle and transport-related manufacturers.

The outputs needed to target these audiences vary (Additional File 4 - Fig. 4).

## Discussion

Air pollution is the regarded as the largest single environmental risk to health, with seven million deaths globally attributed annually [39]. Environmental health and sustainable development goals identified by the WHO highlight the need for effective preventative interventions to protect children from adverse environmental exposures including air pollution [3]. In the UK, road traffic represents the largest contributor to air pollution in urban areas, [40] with the period between 1949 and 2012 seeing a tenfold increase in the distance travelled by the average person in their car [2]. There is an urgent need to identify and quantify the impacts of effective air quality improvement health policy interventions. Such interventions are often costly to implement requiring large alterations in social functioning and urban infrastructure. It is essential therefore that evaluations deliver robust evidence to justify their implementation.

The use of a parallel prospective cohort design enables the CHILL study to deliver the most reliable conclusions to date on exposure-response functions and causal relationships between pollutant exposures and children’s health and lung development. To our knowledge, no previous study has prospectively evaluated a major city-wide air quality mitigation strategy using this design. Like many current research studies, the COVID-19 pandemic has created both operational difficulties in delivering studies, and scientific challenges in disentangling effects of planned interventions from those caused directly from COVID-19 infection and indirectly from attempts to mitigate effects of the pandemic on society. In response to the COVID-19 pandemic, two amendments are made within the study. Firstly, a fifth year of data collection is added to make up for the inability to capture outcome data during school closures and COVID-19 lockdowns. Secondly, where possible the children moving up into secondary school are followed up with a health assessment and collection of the parental questionnaire. By extending data collection by a further year, following children up as they move to secondary schools, collecting evidence of SARS-CoV-2 infection in participants, and collecting contextual data of local impacts of lockdowns, we hope to draw robust conclusions on the effects of implementing the ULEZ on children’s health and development.

With increasing proportions of the world’s population now living in large urban areas commonly far exceeding WHO pollution limit guideline, [3] CHILL study findings will have important implications for the design and implementation of LEZs and Clean Air Zones in the UK, Europe and beyond.

### Electronic supplementary material

Supplementary table for Tables (PDF 38 kb)

Supplementary table for Tables (PDF 38 kb)

Supplementary table for Tables (PDF 38 kb)

Supplementary table for Tables (PDF 38 kb)

## Data Availability

The datasets used and/or analysed during the current study are available from the corresponding author upon reasonable request.

## Abbreviations

ARC: Applied Research Collaboration
AURN: Automatic Urban & Rural Network
BMI: Body mass index
CHILL: Children’s Health in London and Luton
CHS: California Children’s Health Study
CHU9D: Child Health Utility 9 Dimensions questionnaire
CLAHRC: Collaboration for Leadership in Applied Health Research and Care
CMAQ: Community Multiscale Air Quality Modelling System
COVID-19: SARS-CoV-2 Coronavirus
FEV_1_: Forced Expiratory Flow in one second
FVC: Forced Vital Capacity
GPS: Global Positioning Satellite
GP: General Practitioner
HDV: Heavy duty vehicles
HES: Hospital Episode Statistics
HPRU: Health Research Health Protection Research Unit
ICC: Intra-class correlation
ISAAC: International Study of Asthma and Allergy
ISC: Independent Steering Committee
LAQN: London Air Quality Network
LEZ: Low Emission Zone
NHS: National Health Service
NICE: National Institute of Health and Care Excellence
NIHR: National Institute for Health Research
NOx: Nitrogen oxides
NO_2_: Nitrogen dioxide
O_3_: Ozone
PCTU: Pragmatic Clinical Trials Unit
PHE: Public Health England
POA: Primary Organic Aerosol
PM_2.5_: Particulate matter with a diameter of less than 2.5micrograms
PM_10_: Particulate matter with a diameter of less than 10 micrograms
PPI: Patient and public involvement
PMG: Project Management Group
QMUL: Queen Mary University of London
QR: Quick Response code
SOA: Secondary Organic Aerosol
ULEZ: Ultra Low Emission Zone
UKHSA: UK Health Security Agency
WRF: Weather Research and Forecasting model
WHO: World Health Organization
