# Supplementary figures and images for: Investigating the impact of London’s ultra low emission zone on children’s health: children’s health in London and Luton (CHILL) protocol for a prospective parallel cohort study
Source: BMC Pediatr. 2023 Nov 4;23:556. doi: 10.1186/s12887-023-04384-5 (PMC10625305; doi:10.1186/s12887-023-04384-5)

**Figure 1: Study Scheme diagram**


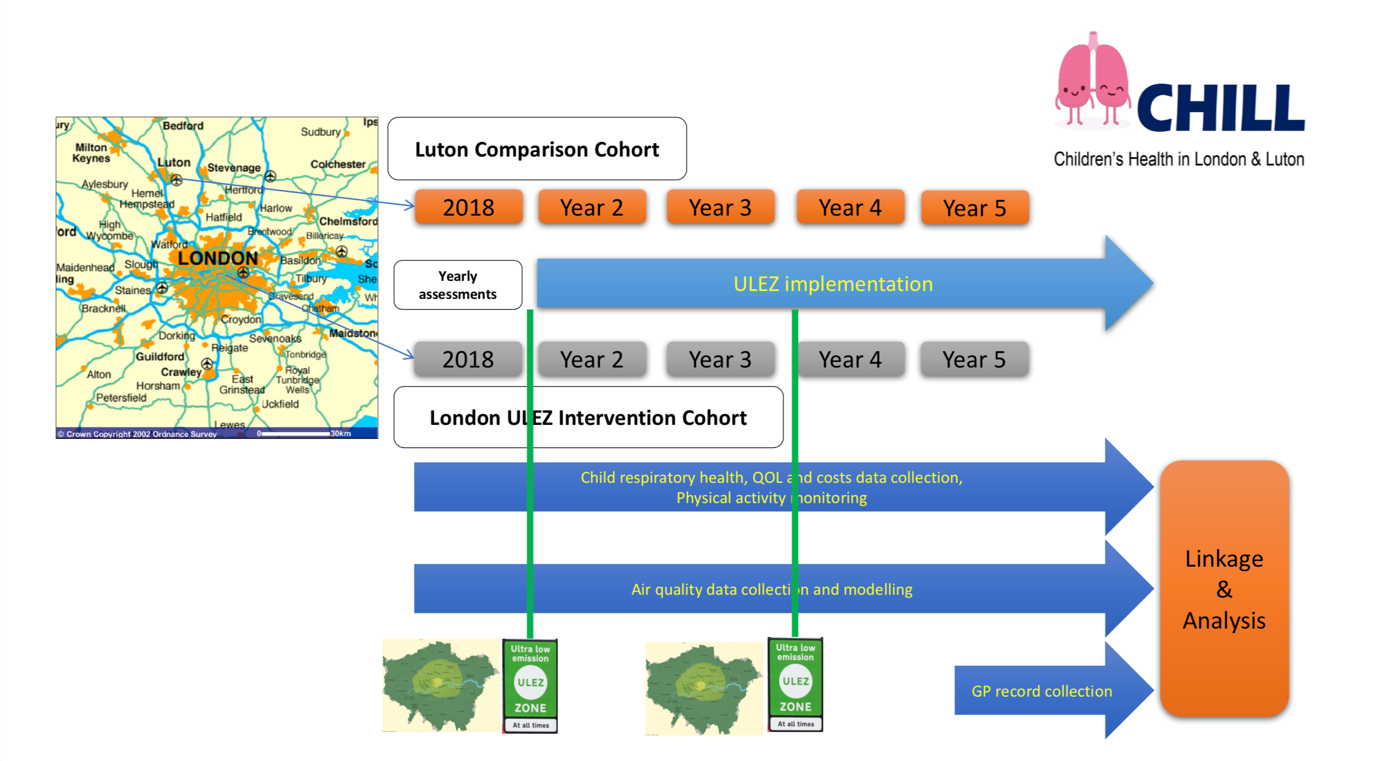

Supplement: Supplementary file 1 — Supplementary table for Tables (PDF 38 kb) [file 12887_2023_4384_MOESM1_ESM.docx]

**Figure 2: ULEZ configuration during phase 1 (2019) and the subsequent extension, scheduled for 2021.**
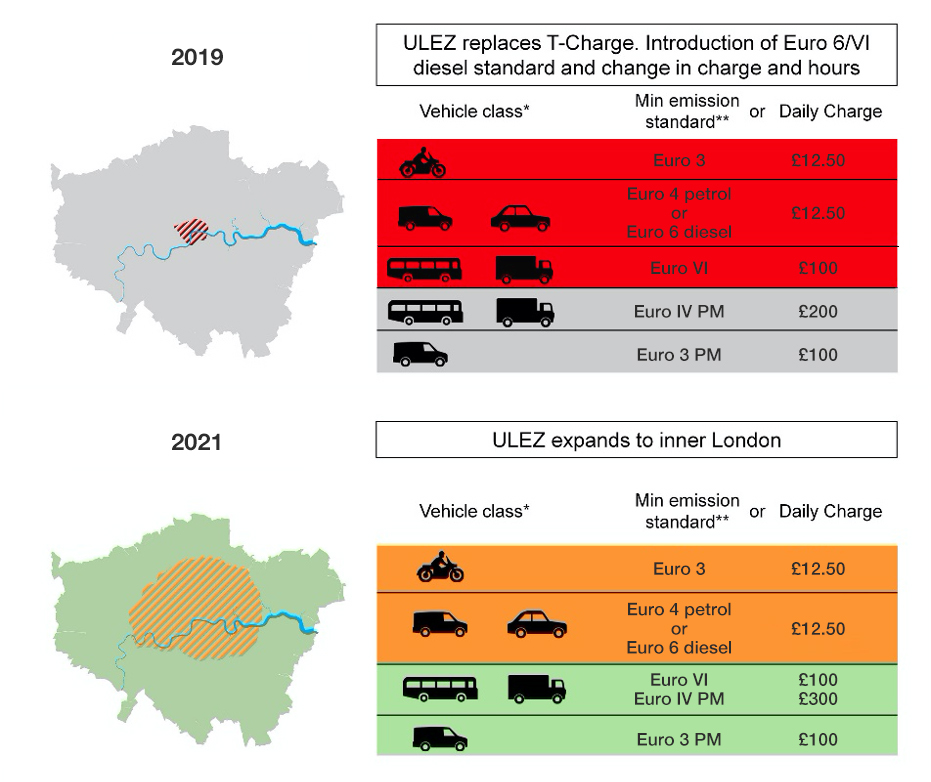

Supplement: Supplementary file 2 — Supplementary table for Tables (PDF 38 kb) [file 12887_2023_4384_MOESM2_ESM.docx]
