# Supplementary material for: Investigating the impact of London’s ultra low emission zone on children’s health: children’s health in London and Luton (CHILL) protocol for a prospective parallel cohort study
Source: BMC Pediatr. 2023 Nov 4;23:556. doi: 10.1186/s12887-023-04384-5 (PMC10625305; doi:10.1186/s12887-023-04384-5)
Supplement: Supplementary file 4 — Supplementary table for Tables (PDF 38 kb) [file 12887_2023_4384_MOESM4_ESM.docx]

**Figure 4: Methods of Dissemination**

| 1) Social media, especially Twitter handles of our institutions to rapidly disseminate succinct information, particularly to the general public, study participants and lay groups.  2) Webinars on websites of our institutions, to provide more detailed summaries of results, with downloads of key documents.  3) Local, national and international television coverage.  4) User friendly study updates and newsletters for participating schools and families  5) Presentations, especially to London and Luton partner organisations including the Greater London Authority (GLA), councils and Health and Wellbeing Boards.  6) Presentations at national and international conferences.  7) Peer reviewed publications targeted to leading medical journals. |
| --- |
